# Effect of *Radopholus similis*, *Pratylenchus araucensis*, *Meloidogyne* spp. and their interaction on *Musa* AAB ‘Dominico Hartón’ seedlings

**DOI:** 10.2478/jofnem-2023-0054

**Published:** 2023-11-24

**Authors:** O. A. Guzmán-Piedrahita, C. Zamorano-Montañez, J. Leguizamon-Caycedo, B. L. Castro-Caicedo, H. D. Lopez-Nicora

**Affiliations:** Programa de Doctorado en Ciencias Agrarias, Universidad de Caldas, Manizales, Caldas, Colombia; Principal researcher in Plant Pathology at Cenicafe, Chinchiná, Colombia, retired; Scientific researcher II in Plant Pathology at Cenicafe, Chinchiná, Colombia, retired; Department of Plant Pathology, The Ohio State University, Columbus, OH, USA

**Keywords:** Host-parasitic relationship, interaction, *Meloidogyne incognita*, *M. arenaria*, *Musa* AAB ‘Dominico Hartón’, *Radopholus similis*

## Abstract

The effect of *Radopholus similis*, *Pratylenchus araucensis*, *Meloidogyne* spp., and their interaction was evaluated in seedlings of *Musa* AAB ‘Dominico Hartón’. The study was conducted in a nursery in Palestina, Caldas department, Colombia. Forty-day-old plantain seedlings were infected separately with 750, 1,500, 2,250 and 3,000 of each species of nematodes/plant. Two experiments were conducted to evaluate the damage of *R. similis*, *P. araucensis*, *Meloidogyne* spp. and the mixture of 750 *R. similis* + 750 *P. araucensis* + 750 *Meloidogyne* spp. compared with the mixture of different proportions (1,500, 2,250 and 3,000 of each species of nematodes). Noninfected plants were included as a control treatment, for a total of 17 treatments in a randomized complete block design with ten replications. Twelve weeks after inoculation, all nematodes, both alone and in combination, reduced (*p* < 0.05) plantain dry root and shoot weight. In two experiments, *R. similis*, *P. araucensis*, and *Meloidogyne* spp. alone, each with a population density of 3,000, reduced (*p* < 0.05) root dry weight by 32.5%, 9.5% and 49%, respectively, and decreased (*p* < 0.05) shoot dry weight by 21.5%, 23%, and 31.5%, respectively, compared to the control. The interaction of nematodes with the lowest population decreased root (33%) and shoot (21%) weight. We conclude that the growth of ‘Dominico Hartón’ seedlings was affected by plant-parasitic nematodes, but the greatest damage occurred with concomitant nematode infection.

In Colombia, plantain [*Musa* AAB (Plantain subgroup)] is grown on more than 400,000 ha with a yield average of approximately 8,000 kg/ha (Agronet, 2020). Annually, this crop generates over 300,000 permanent jobs and is considered a food staple of great socioeconomic importance in small-scale agriculture (Espinal et al., 2006). Plantain is often grown in coffee production regions as part of a cropping sequence. ‘Dominico Hartón’ is the most common plantain variety in Colombia, planted in over 61% of the central coffee growing region. ‘Dominico Hartón’ has a higher level of consumption and a greater commercial value compared to other plantains such as ‘Dominico’, ‘dwarf Hartón’ and ‘Hartón’ (Grisales and Lescot, 1999). In Colombia, under experimental field conditions, *Musa* AAB ‘Dominico Hartón’ can yield between as much as 15,700 kg/ha (Corpoica, 1996) and 23,900 kg/ha, respectively (Guzmán et al., 2012); however, these values are difficult to achieve under commercial field conditions, mainly due to phytosanitary problems – including plant-parasitic nematode damage.

To understand the effect of plant-parasitic nematodes on *Musa*, several studies evaluated the individual effects of different nematodes associated with this crop (Tenente et al., 2008; Coyne et al., 2013). For example, *Radopholus similis* (burrowing nematode), considered the most important nematode of banana and plantain worldwide (Sikora et al., 2018), and *Pratylenchus coffeae* (lesion nematode) are migratory endoparasites that cause lesions in the cortical root cells, affecting water and nutrient absorption and reducing fresh root and shoot weight (Davide and Marasigan, 1985; Sikora et al., 2018). Root weight reduction was reported in plants of *Musa* AAA ‘Grand Naine’ (Moens et al., 2003) and ‘Poyo’ (Fallas et al., 1995) infested with *R. similis*. On the other hand, when *Musa* AAA ‘Grand Naine’ plants were infested with *P. coffeae*, there was no reduction in root or shoot weight (Moens et al., 2006). Recently, Múnera et al. (2009) reported that *Pratylenchus araucensis* n. sp., was found to be associated with *Musa* AAB ‘Hartón’ in Northeast Colombia, but the effects of *P. araucensis* n. sp. on plant growth and yield of *Musa* AAB ‘Hartón’ or ‘Dominico Hartón’ have yet to be evaluated.

The effect of root-knot nematodes, *Meloidogyne* spp., on *Musa* plants has also previously been evaluated (Moens et al., 2005). The species of *Meloidogyne* associated with banana and plantain are *M. incognita*, *M. arenaria*, *M. javanica* and *M. hapla* (De Waele and Davide, 1998; Sikora et al., 2018), of which *M. incognita* and *M. javanica* are the most frequently reported (Moens et al., 2006). Jonathan and Rajendran (2000) in India, and Tenente et al. (2008) in Brazil, inoculated *Musa* cultivars belonging to the AAA, AAAA, AAB, and AAAB genomic groups with *M. incognita* and reported a reduction in root and shoot weight. In Colombia, Jaraba et al. (2008), Navarro et al. (2010) and Riascos et al. (2019), using morphological and morphometric analysis and molecular techniques, reported mixed populations of *M. acrita*, *M. arenaria, M. hispanicam*, *M. incognita* and *M. javanica* associated with *Musa* AAB and AAA. Guzmán-Piedrahita et al. (2020), on the other hand, infested seedlings of plantain cultivar ‘Dominico Hartón’ with a mixture of *M. incognita* and *M. arenaria* (1:7 ratio) and reported a mean reduction of 39% in dry shoot weight and 47% in root weight.

Commercial fields are usually infested with polyspecific communities of plant-parasitic nematodes that can concomitantly infect roots and corms of musaceae plants, resulting in growth reduction and yield losses, and possibly causing more damage than single-species infection (De Waele and Davide, 1998; Moens et al., 2006; Herradura et al., 2012; Sikora et al., 2018). There is little information about the combined damage of multiple plant-parasitic nematodes on *Musa* growth; therefore, it is important to understand their simultaneous effect. For example, Coyne et al. (2013) infested *Musa* AAB ‘Agbagba’ with 1,000 *R. similis*, *P. coffeae*, *Helicotylenchus multicinctus* and *Meloidogyne* spp. individually and with a combination of the four species. They observed that root weights were affected only when *R. similis* infested plants. Similarly, Barekye et al. (2000) examined the pathogenicity of *R. similis* and *H. multicinctus* in pure and mixed populations in tissue-cultured banana plantlets, and reported that *R. similis,* both alone and mixed with *H. multicinctus,* significantly reduced root weight.

*Musa* crops are affected by several plant-parasitic nematodes (De Waele and Davide, 1998; Sikora et al., 2018). In Colombia, however, there is little information of the damage caused by *R similis*, *P. araucensis* and *Meloidogyne* spp. on *Musa* AAB (Plantain subgroup) cv. ‘Dominico Hartón’ plants. The objective of this study was to evaluate the individual effect of different populations of *R. similis*, *P. araucensis*, and *Meloidogyne* spp. and their interaction on *Musa* AAB (Plantain subgroup) cv. ‘Dominico Hartón’ seedlings.

## Materials and Methods

### Study site

The study was conducted in a nursery, under field conditions at the Montelindo Research Farm of Universidad de Caldas located in Palestina, Colombia (5° 05′N and 75° 40′W), with an altitude of 1,050 m, annual average temperature of 23 °C, relative humidity of 74% and annual rainfall of 2,100 mm. Samples were processed in the Nematology Laboratory of Universidad de Caldas in Manizales, Colombia, located 35 km northeast of Montelindo Research Farm.

### Substrate preparation

The substrate used in the experiment consisted of a mixture of soil and sand at a 3:1 ratio, resulting in a sandy loam mixture (80% sand, 14% silt and 6% clay) with pH of 4.9, base content of Ca 3.87, Mg 1.53 and K 0.53 cmol(^+^)/kg, and P 17, Fe 165, Mn 26.74, Zn 10.75, Cu 4.18, S 41.48 and Bo 0.04 mg/kg. The substrate was treated with 50 g/m^2^ of Dazomet (Basamid^®^), watered to saturation and covered with polyethylene film for 45 days. After soil treatment, subsamples of the soil were processed (Jenkins, 1964) to verify no nematodes were detected in sterilized soil.

### Seedling production

From a year-old plantain crop, 100 young suckers (i.e., corms) were collected with an average weight of 1 kg. Suckers were cleaned with a sanitary technique based on Loos and Loos (1960) and modified by Guzmán et al. (2012). The technique for obtaining clear cream-colored suckers consisted of peeling corms with a clean knife and removing necrotic tissue. Suckers were submerged in spinetoram (Exalt, Corteva Agriscience, Indianapolis, IN, USA) and clorpirifos ethyl (Lorsban, Corteva Agriscience, Indianapolis, IN, USA) solutions of 0.5 ml each/L to prevent damage by insects and were further subjected to the stem fragments technique developed by Moïse (2003) with modifications. Suckers were sown on greenhouse beds, raised 60 cm from the floor, on previously sterilized substrate. Finally, suckers were covered with a layer of sterile substrate 2 to 4 cm thick.

Plants were fertilized every 15 days from the second week after transplant with 6 g of diammonium phosphate (NH_4_ 18%, P_2_O_5_ 48%) and potassium chloride (K_2_O 60%), based on soil analysis and recommendations (Lardizábal and Gutiérrez, 2006). An automatic irrigation system delivered water for 10 minutes twice a day to maintain a soil moisture level of 60% of field capacity.

Fifty days after planting, young suckers with at least three leaves were carefully extracted and roots were removed with a clean knife. Shoots were cut 5 cm from the sucker base. Suckers were weighed and planted in black plastic bags (20 × 32 cm) filled with five kg sterilized substrate. Plastic bags containing seedlings were placed on tables at 50 cm from the ground to avoid contamination. A dark mesh providing 60% shade was placed 180 cm above the tables.

### Nematode infestation and population density

The *M. arenaria* + *M. incognita* (7:1 ratio) mixture population (hereafter referred to as *Meloidogyne* spp.), which was previously characterized with morphological and molecular techniques (Guzmán-Piedrahita et al., 2020), was maintained on *Musa* AAB ‘Dominico Hartón’ plants at Montelindo Research Farm and used in this study. *Radopholus similis* was extracted from roots of *Musa* AAB ‘Dominico Hartón,’ available at Montelindo Research Farm. *Pratylenchus araucensis* was extracted and characterized from *Musa* AAB ‘Dominico Hartón’ roots from a commercial farm in San José, Caldas department, Colombia, at an altitude of 1,440 m. Both *R. similis* and *P. araucensis* were reared on carrot discs (Speijer and De Waele, 1997). Nematodes from infected roots and carrot discs were extracted by macerating the tissue with a blender (Taylor and Loegering, 1953) and separating with the sieving, decanting and centrifugal sucrose flotation technique (Jenkins, 1964). Baermann funnels were used to recover live nematodes (Ayoub, 1981). Recovered nematodes were placed in a 200 ml beaker and suspended in 100 ml tap water, with air was supplied using an air pump (Elite 799, 120 V, 60 Hz, 1.5 W and 1.0 P.S.I.) to homogenize and oxygenate the nematode suspension. The numbers of *R. similis* and *P. araucensis* were counted under the stereo microscope (Unico, Model ZM181HF) at 40×. For samples containing *Meloidogyne* spp., only the number of eggs and second-stage juveniles (J2) were counted.

Forty days after planting, plantain seedlings with uniform height were infested with either 750, 1,500, 2,250 or 3,000 *R. similis*/plant by delivering the nematode inoculum suspension to drench the area around the seedling roots. With the same population densities, plantain seedlings were inoculated with either 750, 1,500, 2,250 or 3,000 *P. araucensis* and *Meloidogyne* spp. alone; the mixture of 750 *R. similis* + 750 *P. araucensis* + 750 *Meloidogyne* spp.; or the mixture of the same species, but in different proportions (1,500, 2,250 and 3,000 of each species of nematode). Noninfested plants were included as control, for a total of 17 treatments under a completely randomized block design with ten replicates. The experiment was repeated, with experiment 1 conducted between November 2018 and April 2019 and experiment 2 between January and July 2019.

### Data collection

Twelve weeks after inoculation, we recorded plant height (distance in cm from the ground level to the base of the flag leaf); leaf number; functional roots (%); the total number of primary roots originating from the surface of the sucker; and the number of primary roots showing lesions by nematodes. Necrosis of functional roots caused by each nematode was evaluated and expressed in percentage, following the method proposed by Carlier et al. (2003). *Meloidogyne* spp. gall index (%) was also recorded (Castaño-Zapata, 1989). Nematode population density was obtained from the soil and plant roots (Taylor and Loegering, 1953) and used to calculate the nematode reproduction factor (*Rf* = final population [in soil and roots]/initial population [inoculation levels]). Finally, roots and shoots were placed in paper bags and dried in an oven (Binder Tuttlingen, Germany) for 8 days at 90 °C. Mean dry root and shoot weight (g)/plant were then recorded.

### Data analysis

Analysis of variance (ANOVA) was used to test for differences in mean root and shoot dry weight, plant height, root necrosis, and leaf number per treatments among inoculum levels, and each treatment were compared to the noninfested plants (control) using Dunnett's test at *p* = 0.05 level. Functional roots were subjected to ANOVA and means separation was done using Tukey's Honest Significant Difference test at *p* = 0.05 level. The relationship between final nematode population densities and root necrosis were examined using Pearson's correlation, as well as the relationship between *Meloidogyne* spp. gall index and final nematode population was assessed using Pearson's correlation coefficient. Data analyses of each experiment were conducted separately. All statistical analyses were performed in R, version 4.2.2 (R Core Team, 2022).

## Results

### Individual effect of plant-parasitic nematodes

**Figure 1:**
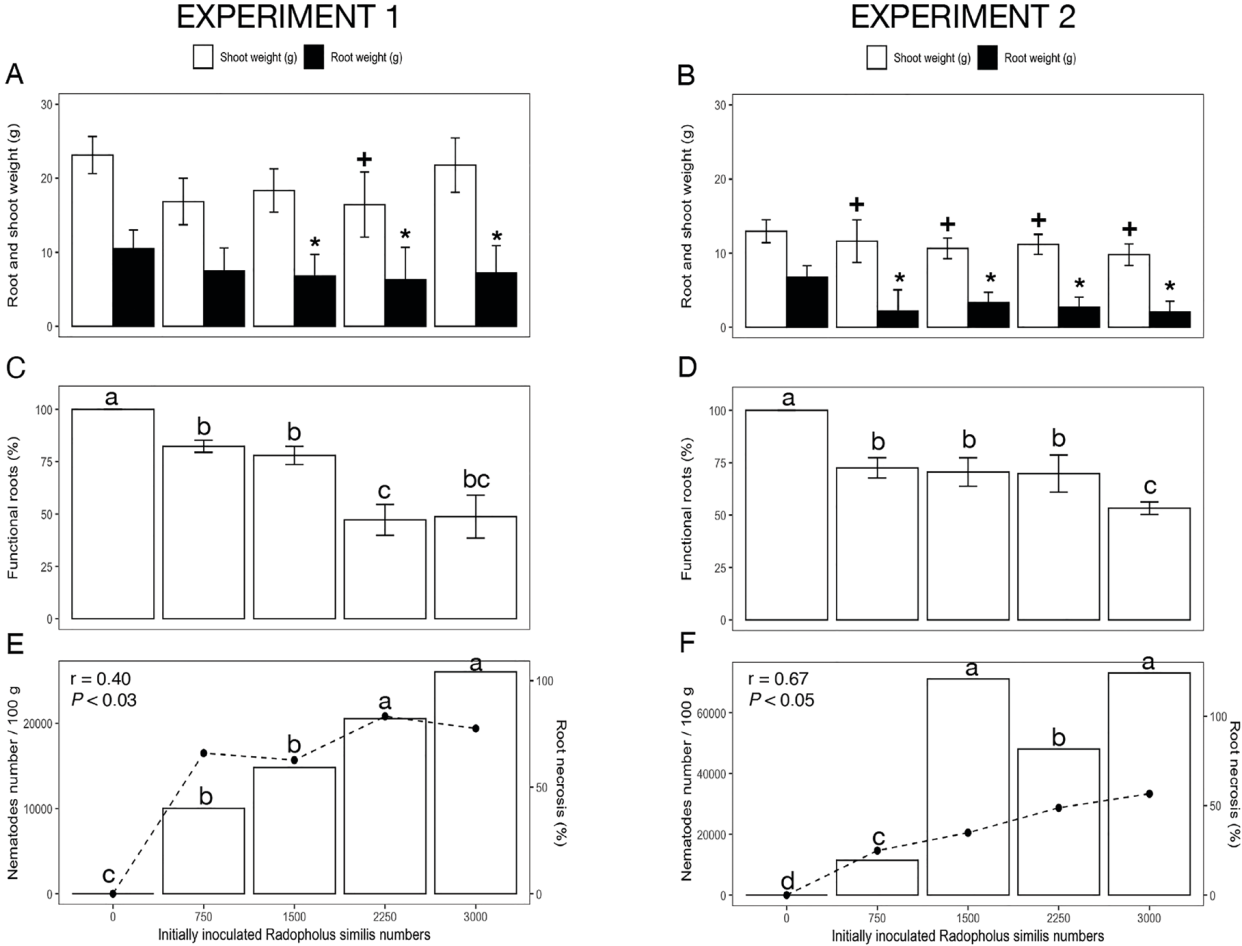
Effect of *Radopholus similis* populations on *Musa* AAB (Plantain subgroup) ‘Dominico Hartón’ seedlings during November 2018 – April 2019 (Experiment 1) and January–July 2019 (Experiment 2). A, B: Root and shoot biomass (g dry weight), (*) indicates differences (*p* < 0.05) in root weight, (+) shoot weight compared to the noninfected control, according to Dunnett's test. C, D: Functional roots (%), different letters indicate difference (*p* = 0.05), using Tukey's test; E, F: Nematode population (nematode number/100 g fresh weight), indicated by line, and root necrosis (%) indicated by bars; and the associated Pearson correlation (r) and probability (p).

In both experiments, 12 weeks after plant inoculation, *R. similis* reduced (*p* < 0.05) dry root and shoot weight as the nematode population density increased (Figs. 1A,B, respectively). Compared to non-infested plants, on average, 3,000 *R. similis* reduced shoot weight by 18% and root weight by 26%, in experiment 1, and by 25% and 39%, respectively, in experiment 2 (Fig. 1A, B).

**Figure 2:**
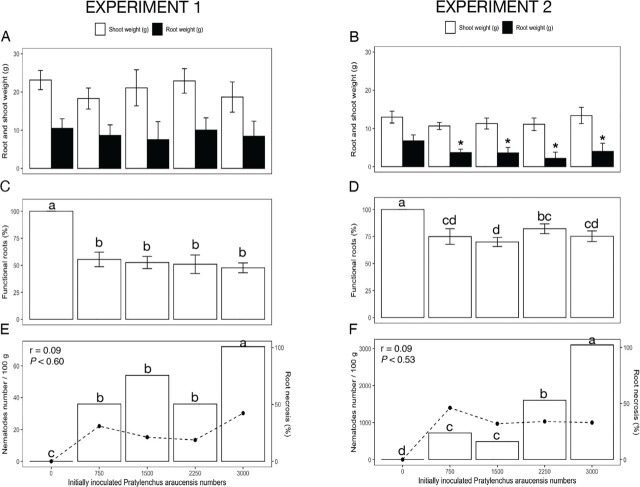
Effect of *Pratylenchus araucensis* populations on *Musa* AAB (Plantain subgroup) ‘Dominico Hartón’ seedlings during November 2018 – April 2019 (Experiment 1) and January–July 2019 (Experiment 2). A, B: Root and shoot biomass (g dry weight), (*) indicates differences (*p* < 0.05) in root weight, (+) shoot weight compared to the noninfected control, according to Dunnett's test. C, D: Functional roots (%), different letters indicate difference (*p* = 0.05), using Tukey's test; E, F: Nematode population (nematode number/100 g fresh weight) indicated by line, and root necrosis (%) indicated by bars; and the associated Pearson correlation (r) and probability (p).

**Figure 3:**
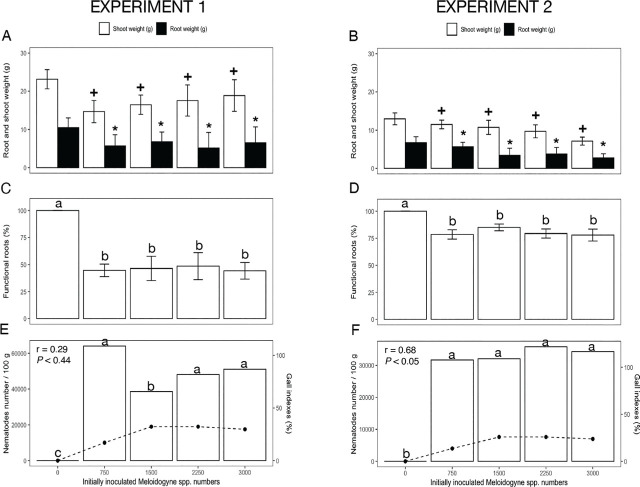
Effect of *Meloidogyne arenaria* + *M. incognita* (7:1 ratio) populations on *Musa* AAB (Plantain subgroup) ‘Dominico Hartón’ seedlings during November 2018–April 2019 (Experiment 1) and January–July 2019 (Experiment 2). A, B: Root and shoot biomass (g dry weight), (*) indicates differences (*p* < 0.05) in root weight, (+) shoot weight compared to the noninfected control, according to Dunnett's test. C, D: Functional roots (%), different letters indicate significant difference (*p* = 0.05), using Tukey's test; E, F: Nematode population (nematode number/100 g fresh weight) indicated by line, and gall indexes (%) indicated by bars; and the associated Pearson correlation (r) and probability (p).

In the first experiment, 3,000 *P. araucensis* did not reduce shoot and root weight, but in experiment 2, root weight was reduced (*p* < 0.05) by 43% (Figs. 2A,B). A stimulatory effect on root and shoot weight was observed with populations from 1,500 to 2,250 of *P. araucensis* in experiment 1. In experiment 2, this effect was observed with 3,000 nematodes (Figs. 2A,B). On the other hand, in experiment 1, various population densities of *Meloidogyne* spp. decreased (*p* < 0.05) dry shoot weight, at all inoculation rates, by 18% to 37% (relative to non-inoculated), and reduced dry root weight by 38% to 46% (Fig. 3A). In experiment 2, dry shoot weight decreased (*p* < 0.05) for all inoculation rates relative to non-inoculated between 11% and 45%, and dry root weight decreased by 18% to 60% (Fig. 3B).

In both experiments, primary roots originating from the surface of the sucker were infected by *R. similis* (Table 1). Plants infested with *R. similis* had up to 63% of their roots originating from the sucker, showing lesions in experiment 1 when compared to non-infested plants. However, *P. araucensis* caused less than 31% of the damaged roots in experiment 2 (Table 1). *Meloidogyne* spp. Caused 70% of roots originating from the sucker to show lesions in experiment 1, and 69% in experiment 2 (Table 1).

**Table 1.** Plantain growth of (height and number of leaves) and number of root bases (places where roots attach to the corm) on the sucker showing lesions by nematodes on *Musa* AAB ‘Dominico Hartón’ inoculated with varying initial densities of *Radopholus similis*, *Pratylenchus araucensis*, *Meloidogyne* spp. and the mixture of 750 *R. similis* + 750 *P. araucensis* + 750 *Meloidogyne* spp. and the mixture of other proportions (1,500, 2,250 and 3,000 of each species of nematodes).

**EXP**	**Nematode population**	***Radopholus similis***				***Pratylenchus araucensis***				***Meloidogyne* spp.**				**Concomitant nematode infestation**			
			
		**Height (cm)**	**Leaf (#)**	**Root base (#)**		**Height (cm)**	**Leaf (#)**	**Root base (#)**		**Height (cm)**	**Leaf (#)**	**Root base (#)**		**Height (cm)**	**Leaf (#)**	**Root base (#)**	
			
				**Lesion**	**Total**			**Lesion**	**Total**			**Lesion**	**Total**			**Lesion**	**Total**
1	Control^y^	43 a^x^	5 a	0	27	43 a	5 a	0	27	43 a	5 a	0	27	43 a	5 a	0	27
	750	37 b	6 a	18	25	36 b	5 a	13	21	36 b	5 a	14	23	35 b	5 a	16	20
	1,500	37 b	5 a	17	21	37 b	5 a	16	24	38 b	5 a	14	20	38 a	5 a	18	23
	2,250	37 b	6 a	18	20	40 b	5 a	17	26	40 b	5 a	16	23	37 a	4 a	19	21
	3,000	38 b	5 a	20	24	37 b	5 a	14	22	40 b	6 a	15	20	32 a	5 a	18	20
2	Control	29 a	6 a	0	26	29 a	6 a	0	26	29 a	6 a	0	26	29 a	6 a	0	26
	750	26 b	4 b	11	21	28 b	4 b	5	19	31 a	4 b	9	27	25 b	4 b	13	20
	1,500	25 b	4 b	14	21	28 ab	4 b	8	20	30 a	4 b	5	23	27 b	4 b	16	19
	2,250	28 ab	4 b	17	22	27 b	4 b	6	19	27 a	4 b	5	18	25 b	4 b	18	21
	3,000	25 b	4 b	15	18	31 a	4 b	8	25	23 b	4 b	6	15	25 b	4 b	18	21

x Means in the same column followed by the same letter do not differ significantly (*p* ≤ 0.05) according to Dunnett's test.

y One single control with ten reps was used for each one nematode.

Population densities of *R. similis* and *Meloidogyne* spp. Were related in damage severity to the root system of *Musa* AAB ‘Dominico Hartón’ plants. Relative to non-infested plants (which did not show dead roots), the lowest population density of *R. similis* reduced functional root weight by more than 20% in both experiments 1 and 2, and the highest population densities caused a 50% reduction in functional root weight (Figs. 1C,D).

In both experiments, *P. araucensis* reduced functional root weight between 19% and 39% compared to non-infested plants (Figs. 2C,D). All population densities of *Meloidogyne* spp. affected (*p* < 0.05) plant roots, causing between 47% and 54% loss of functional roots in experiment 1 (Fig. 3C). In experiment 2, functional roots were reduced by 22% (Fig. 3D). In both experiments, plants infested with a nematode population density higher than 1,500 eggs and juveniles/plant caused a gall index ranging from 38% to 50% (Figs. 3E,F).

Only in experiment 2 plants infected with *R. similis*, *P. araucensis* or *Meloidogyne* spp. have fewer (*p* < 0.05) leaves compared to noninfected plants (Table 1). In experiment 2, noninfected plants had 9.5% more leaves (*p* < 0.05) than those infested with *R. similis* and *P. araucensis* (Table 1).

**Figure 4:**
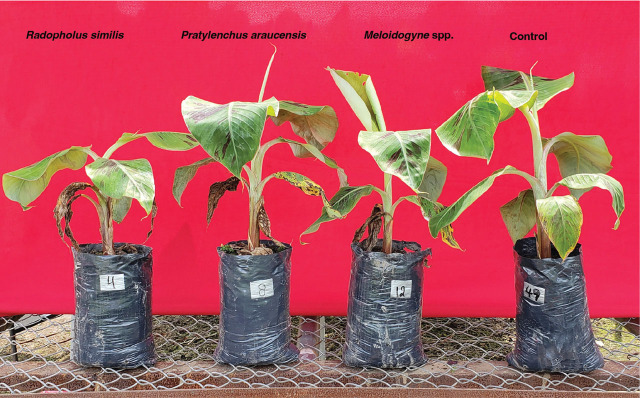
Effect of the highest population of plant-parasitic nematodes on plant height and number of leaves of *Musa* AAB (Plantain subgroup) ‘Dominico Hartón’ seedlings, during January–July 2019. Plants infected with 3,000 nematodes/plant, from left to right: *Radopholus similis*, *Pratylenchus araucensis, Meloidogyne* spp., and mixed populations of 3,000 *R. similis* + 3,000 P. araucensis + 3,000 *Meloidogyne* spp., and noninfected plant (control).

In experiment 1, *Meloidogyne* spp. decreased (*p* < 0.05) plant height by over 11% compared to non-infested plants. In experiment 2, however, plant height was reduced (*p* < 0.05) by over 20%, but only when infested with 3,000 *Meloidogyne* spp. (Table 1, Fig. 4).

On average, at all *R. similis* inoculum densities, an Rf value > 1 was observed. In experiment 1, from the lowest to the highest initial population density, *R. similis* increased to 10,882 and 22,790 individuals (Rf = 14.44 and 7.59) (Fig. 1E). Reproduction was similar in experiment 2, with final population densities of 6,999 and 30,159 individuals (Rf = 9.33 and 10.05) for the lowest and highest population density, respectively (Fig. 1F). In both experiments, the RF for *P. araucensis* was < 1 (Figs. 2E,F). In experiment 1, the final population of *Meloidogyne* spp. increased to 64,723 and 63,244 (Rf = 86.29 and 21.08) from the lowest to the highest initial population densities, respectively (Fig. 3E). In experiment 2, by contrast, final population densities had values of 24,088 and 17,010 (Rf = 32.12 and 5.67) from the lowest to the highest initial populations, respectively (Fig. 3F).

### Concomitant effect of plant-parasitic nematodes

**Figure 5:**
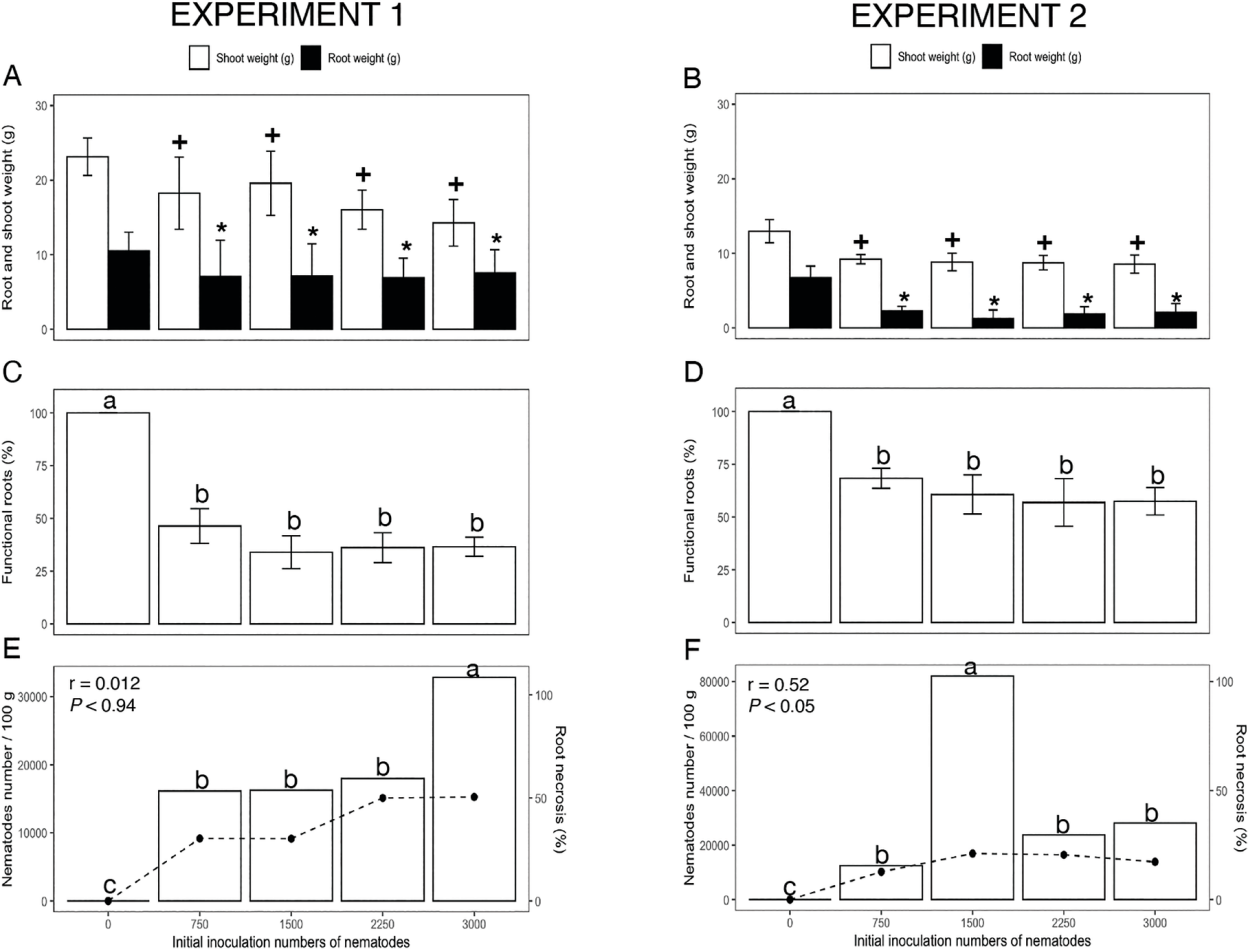
Concomitant effect of *Radopholus similis*, *Pratylenchus araucensis* and *Meloidogyne* spp. populations on *Musa* AAB (Plantain subgroup) ‘Dominico Hartón’ seedlings during November 2018 – April 2019 (Experiment 1) and January–July 2019 (Experiment 2). A, B: Root and shoot biomass (g dry weight), (*) indicates differences (*p* < 0.05) in root weight, (+) shoot weight compared to the noninfected control, according to Dunnett's test. C, D: Functional roots (%), different letters indicate difference (*p* = 0.05) using Tukey's test; E, F: Nematode population (nematode number/100 g fresh weight) indicated by line, and root necrosis (%) indicated by bars; and the associated Pearson correlation (r) and probability (p).

Simultaneous infection of *R. similis, P. araucensis* and *Meloidogyne* spp. caused a decrease (*p* < 0.05) in dry root and shoot weight of plantain seedlings. Compared to non-infected plants, a mixture of 750 *R. similis* + 750 *P. araucensis* + 750 *Meloidogyne* spp. reduced dry root weight by 33% and dry shoot rate by 21% in experiment 1 (Fig. 5A). In experiment 2, the mixture of nematodes decreased dry root weight by 29% and and dry shoot weight by 72% (Fig. 5B). In experiment 1, the mixture of *R. similis, P. araucensis* and *Meloidogyne* spp. decreased functional roots between 51% and 62% (Fig. 5C); in experiment 2, there was an average of 36% functional roots lost (Fig. 5D). Furthermore, roots originating from the sucker exhibited lesions ranging from 65 to 80% when simultaneously infested with nematodes (Table 1).

In both experiments, more necrotic roots (*p* < 0.05) were associated with the two highest nematode population densities, with 55% at 2,250 and 3,000 at 66%. At the lowest population density, root necrosis ranged between 37% and 40% (Figs. 5E,F). Gall index was less than 20% for all inoculation levels (data not shown). In experiment 1, the mixture of nematodes increased to 17,076 (Rf = 7.58 and 4.06) from the lowest (mixture of 750 *R. similis* + 750 *P. araucensis* + 750 *Meloidogyne* spp.) and 36,591 individuals from the highest (mixture of 3,000 *R. similis* + 3,000 *P. araucensis* + 3,000 *Meloidogyne* spp.) initial populations (Fig. 5E). In experiment 2, the final population of mixture of nematodes consisted of 6,573 and 13,710 individuals (Rf = 2.92 and 1.52) for the lowest and highest population densities, respectively (Fig. 5F).

The combination of *R. similis, P. araucensis* and *Meloidogyne* spp. decreased (*p* < 0.05) leaf number only in experiment 2. Noninfected plants had two additional leaves compared to infected plants (Table 1). In both experiments, this combination of nematodes also reduced (*p* < 0.05) plant height; infected plants were 5 to 10 cm shorter than noninfected plants in experiment 1, and 2 to 5 cm shorter in experiment 2 (Table 1).

## Discussion

### Individual effects of nematode infestation Musa

AAB (Plantain subgroup) ‘Dominico Hartón’ plant development was affected by *R. similis*, *P. araucensis* and *M. arenaria + M. incognita* in nursery conditions 12 weeks after plant inoculation. In the two experiments, plant-parasitic nematodes decreased dry root and shoot weight as inoculum densities were increased. Similar results were reported in plantain by Coyne et al. (2013), in which *Musa* AAB ‘Agbagba’ was infested with 1,000 *R. similis*/pot, and Marin et al. (2000), who infested *Musa* AAB ‘False Horn’ with 200 *R. similis*/plant. A lower fresh shoot weight was observed on infected plants.

In banana, where studies are more prolific, similar results had been previously reported. Fallas et al. (1995) reported lower fresh root and shoot weight 12 weeks after infecting *Musa* AAA ‘Poyo’ plantlets with 100 *R. similis*/plant. They also found greater differences in roots than in shoots, similar to the results reported in our current study. Similarly, under controlled experimental conditions, Hahn et al. (1996) infested 100 or 300 *R. similis*/plant from Uganda, Sri Lanka and Indonesia on *Musa* AAA (Cavendish subgroup) ‘Poyo’ plantlets and 200 *R. similis*/plant on (Ibota group) ‘Yangambi’, and found lower root and fresh shoot weight in those plants.

Moens et al. (2003) also infected banana plants of *Musa* AAA ‘Grand Naine’ with 4,000 *R*. *similis*/pot. Moens et al. (2005) also infected *Musa* AAAA ‘FHIA-17’ with 421 *R. similis* and reported fresh root weight reductions of 68% and shoot weight reductions of 24%. From these observations, Fallas et al. (1995) and Hahn et al. (1996) determined that the reduction of root weight in plants infested with *R. similis* was a good indicator of its pathogenicity on banana and plantain plants. Results obtained in nursery conditions in our research study supported this conclusion.

Múnera et al. (2009) reported *Pratylenchus araucensis* n. sp. parasitizing *Musa* AAB ‘Hartón’ plants. Our study is the first to evaluate the effect of this nematode species on this *Musa* AAB cultivar. In this study, plants of *Musa* AAB ‘Hartón’ infected with 750 nematodes/plant of *P. araucensis* had a decreased dry root and shoot weight. In another study, Van den Bergh et al. (2002) investigated the reaction of selected Vietnamese *Musa* germplasm to *P. coffeae* and found that *P. coffeae* decreased in shoot and root weight. However, no effect on plant height, number of standing leaves or the girth of the plants was observed. Similarly, Brentu et al. (2004) and Van den Bergh et al. (2006) reported that *P. coffeae* did not affect plant height or number of standing leaves of *Musa* plants at harvest.

In our study, *P. araucensis* had less reproductive capacity and caused less root damage. Plausible explanations may relate to nematode biology, especially a higher male:female ratio, and, possibly, the nematodes requiring more time to reproduce and cause damage.

Multiple studies have shown *Meloidogyne* spp. causing dry root and shoot weight reduction in *Musa* plants. For example, Tenente et al. (2008) inoculated *Musa* plants with *M. incognita* race 4 and reported a reduction in root weight across different cultivars: ‘Ambrosia’, ‘Bucaneiro’, ‘Grand Naine’, ‘Thap Maceo’, ‘PA-4219’, ‘Prata Zulu’, and ‘YB-4207’. However, when they compared *Musa* plants infected with *M. incognita* race 1 to noninfected plants, no difference in root weight and plant height was observed.

Moens et al. (2005), infected *Musa* AA cultivars *Musa ac. malaccensis*, ‘Niyarma Yik’ and ‘Tjau Lagada,’ with *M. incognita* and reported fresh root weight reductions of 44%, 43% and 28%, respectively. Moens et al. (2006) also reported a 9% reduction in fresh root weight in *Musa* AAA ‘Grand Naine’ plants infected with *M. incognita* compared to those inoculated. Similarly, Davide and Marasigan (1985) and Jonathan and Rajendran (2000) infected ‘Giant Cavendish’ and ‘Poovan’ cultivars with 1,000 J2 of *M. incognita*/plant and reported reduction up to 31% in plant growth. Root and shoot reduction similar to that reported in our study have also been reported elsewhere (Adiko, 1989; Sudha & Prabhoo, 1983).

In our study (experiment 1), *R. similis* did not affect plant height and leaves number of *Musa* AAB ‘Dominico Hartón.’ Similar results were reported by Hahn et al. (1996) on *Musa* AAA ‘Poyo’ and ‘Yangambi’ plants; by Marin et al. (1999) on *Musa* AAA ‘Grand Naine’ plants; by Barekye et al. (2000) on *Musa* AAA-EA ‘Kisansa’ plants; and by Marin et al. (2000) on *Musa* AAB ‘False Horn’.

Under controlled conditions, Fallas et al. (1995) evaluated reproductive fitness and pathogenicity of eight *R. similis* populations (100 nematodes/plant) on *Musa* AAA ‘Poyo’ plantlets and reported that plant height did not correlate with nematode reproduction, regardless of the nematode population. In a pot experiment, Barekye et al. (2000) reported that *Musa* ‘Kisansa’ plants infected with *R. similis* did not affect the height, growth or the number of functional leaves in *Musa* ‘Kisansa’ plants. Fallas et al. (1995) and Hahn et al. (1996) also reported that *R. similis* did not affect plant height.

In our study, all *Meloidogyne* spp. similar to the ones reported in different studies affected plantain height and leaf number. For example, Jonathan and Rajendran (2000) reported significant reduction in plant pseudostem girth, leaf number, total leaf area, root length and weight in banana ‘Poovan’ plants infested with either 1,000 or 10,000 J2 of *M. incognita*/kg soil. However, when Tenente et al. (2008), in greenhouse conditions, infected various *Musa* cultivars with 15,000 eggs and juveniles of *M. incognita* (race 4), they reported no difference in plant height between infected and noninfected plants for any cultivar. Sudha and Prabhoo (1983) reported poor growth, discoloration, and yellowing of leaves on banana ‘Palayathondan’ heavily infected with *M. incognita*.

Nursery plants in experiment 2 presented lower growth parameters relative to experiment 1. Seedlings in experiment 2 were affected by the herbicide clomazone (Sargent) at 6 weeks after they were infested with nematodes. Herbicide was applied on a crop close (5 m) to the plantain nursery. The product label recommends a minimum safety strip of 10 m for land application. The phytotoxic action of the herbicide involves biosynthetic inhibition of pigments involved in photosynthesis. Consequently, plants showed leaf whitening or yellowing due to the absence of chlorophyll. Symptoms were observed at 3 weeks on plantain seedlings and some weeds around the experiment area.

In our study, *R. similis* infected roots that originated from the plantain's sucker. Similar results were reported by Blomme et al. (2001) when *R. similis* was delivered in the planting hole of six *Musa* genotypes. Nematodes begin colonizing in the first 10 cm adjacent to the corm, producing 50% necrotic root tissue in sword sucker-derived ‘Agbagba’ and ‘Obino l’Ewai’ plantain. In Honduras, Viaene et al. (2003) reported that *R. similis* was responsible for lesions in 85% of the root bases in *Musa* AAB ‘Cuerno’. Destruction of the root base by *R. similis* probably explains why infection with this nematode normally results in plants toppling (Barekye et al. 2000). Pinochet (1977) also reported the lesions caused by *R. similis* were observed closer to the sucker.

Plants infected with *R. similis* showed root necrosis and less root weight, similar to results obtained by Viaene et al. (2003) and Marin et al. (2000), who reported 49% root necrosis in plantain *Musa* AAB ‘Cuerno’ and 60% in ‘False Horn’ to *R. similis*. Also, in southern Nigeria, root necrosis percentage, under field conditions averaged 15% for plant-parasitic nematodes (Speijer et al., 2001). These results agreed with Marin et al. (1989) and Marin et al. (2000), which reported that necrosis of primary roots, rather than root weight, was the best indicator of variation in *R. similis* reproduction. Conversely, Hahn et al. (1996) suggested that reduction in plant root weight was the best indicator of pathogenicity.

In our study, *R. similis* population density increased from the various initial nematode populations when compared to uninfected plants. Similar results were reported by Barekye et al. (2000) who infested plants with 1,000 nematodes and obtained an increase of 12,590 (trial 1) and 13,183 (trial 2) nematodes/100 g of roots. Similarly, Marin et al. (2000) reported that plantain ‘False Horn’ and banana cultivars ‘Valery’, ‘Grand Naine’, and ‘Lacatan’ supported greater reproduction of *R. similis* and had the highest percentage of root necrosis among genotypes tested in the greenhouse. In our study, the functional roots were reduced by *R. similis* at each density level of nematodes inoculated/plant.

Moens et al. (2003) found that when plants of *Musa* AAB ‘Grand Naine’ were infested with 2,000 *R*. *similis*/plant, they reached saturation of the root system after 8 weeks. The same authors initially infested 508 *R*. *similis* female in ‘Grand Naine’ plants every 2 weeks over a period of 16 weeks. After a steady increase up to 12 weeks after inoculation, a stabilization or even decrease was observed in *R*. *similis*/100 g of roots, reproductive index, root and shoot weight.

Finally, we found that as nematode numbers increase, root damage increases and dry shoot weight decreases, and plantain infected with lowest population density of *Meloidogyne* spp. had the highest Rf. Adiko (1989) also found a decrease in the reproduction factor of *M. incognita* as the inoculum level increased. Likewise, Jonathan and Rajendran (2000) reported that multiplication of *M. incognita* in terms of Rf trended negative at the initial inoculum level. The highest Rf value (491.1) was observed with an inoculum of 10 J2, but it was as low as 3.3 at the inoculum level of 10,000 J2.

It is known that fewer roots become available with higher initial nematode populations, thus creating crowded conditions that adversely affect the development of nematodes (Davide and Thiantaphyllou, 1967; Gonçalves, 1998). In our study, 12 weeks after inoculation with root-knot nematodes *M. arenaria + M. incognita*, massive galling both on the main and the lateral roots, as well as the prolific feeder roots, were observed at all nematode densities. Similar results were reported by De Waele and Davide (1998) on banana, by Adiko (1989) on *Musa* AAB ‘Horse 1’, and by Van den Bergh et al. (2002) on *Musa* genotypes infected with *M. incognita* and *Meloidogyne* spp. under greenhouse conditions.

### Concomitant effect of combined nematode infestation

The combination of *R. similis, P. araucensis* and *Meloidogyne* spp. caused greater root damage than each nematode alone. The damage caused by the mixture of nematodes was synergistic. Similar results were reported by Coyne et al. (2013), with 1,000 nematodes belonging to four different species (*R. similis*, *P. coffeae*, *H. multicinctus* and *Meloidogyne* spp.) in *Musa* AAB ‘Agbagba’ in Nigeria. In the same study, a combination of the four species led to greater root necrosis than in the noninfested control, while *R. similis* alone was found to reduce root mass to a lower level than for other treatments. In our study, *R. similis* colonized *Meloidogyne* spp. root galls, causing destruction and necrosis of root cells and consequently reducing the number of feeding sites for *Meloidogyne* spp. Davide (1980) also reported that a severe infestation of *R. similis* also led to necrosis of roots, and *M. incognita* failed to infect and survive on dead tissues. Pinochet (1977), Davide and Marasigan (1985), and Speijer et al. (2001) have also previously documented the suppression of *Meloidogyne* spp. in banana roots in the presence of *R. similis*.

In summary, the growth of young suckers (i.e., corms) of *Musa* AAB ‘Dominico Hartón’ is severely affected by the most common plant-parasitic nematodes, *R. similis* and *M. arenaria + M. incognita,* in nursery conditions; it was also found that concomitant nematode infestation produced more damage to plants than single species. Our study represents the first report of *P. araucensis* damage on *Musa* plants under experimental conditions. Findings presented in this study will be fundamental in developing integrated pest management strategies in young suckers (corms) in Colombia.
